# Regional and global antimicrobial susceptibility among isolates of *Streptococcus pneumoniae* and *Haemophilus influenzae* collected as part of the Tigecycline Evaluation and Surveillance Trial (T.E.S.T.) from 2009 to 2012 and comparison with previous years of T.E.S.T. (2004-2008)

**DOI:** 10.1186/s12941-014-0052-2

**Published:** 2014-11-07

**Authors:** Viktorija Tomic, Michael J Dowzicky

**Affiliations:** University Clinic of Respiratory and Allergic Diseases, Golnik 36, 4204 Golnik, Slovenia; Pfizer Inc, Collegeville, PA USA

**Keywords:** Antimicrobial susceptibility, *Streptococcus pneumoniae*, *Haemophilus influenzae*, Tigecycline

## Abstract

**Background:**

We report here on 14438 *Streptococcus pneumoniae* and 14770 *Haemophilus influenzae* isolates collected from 560 centres globally between 2004 and 2012 as a part of the Tigecycline Evaluation and Surveillance Trial (T.E.S.T.).

**Methods:**

MIC testing was performed using broth microdilution methods as described by the Clinical and Laboratory Standards Institute (CLSI) using CLSI-approved breakpoints; US Food and Drug Administration breakpoints were used for tigecycline as CLSI breakpoints are not available.

**Results:**

At least 99% of *S. pneumoniae* isolates globally were susceptible to levofloxacin, linezolid, tigecycline or vancomycin. Penicillin resistance was observed among 14.8% of *S. pneumoniae* and was highest in Asia/Pacific Rim (30.1%) and Africa (27.6%); 23.4% of *S. pneumoniae* isolates were penicillin-intermediate, which were most common in Africa (37.6%). Minocycline susceptibility among *S. pneumoniae* decreased by 20% between 2004-2008 and 2009-2012. High (>98.5%) susceptibility was reported among *H. influenzae* to all antimicrobial agents on the T.E.S.T. panel excluding ampicillin, to which only 78.3% were susceptible. β-lactamase production was observed among 20.2% of *H. influenzae* isolates; 1.5% of isolates were β-lactamase negative, ampicillin-resistant.

**Conclusions:**

*S. pneumoniae* remained highly susceptible to levofloxacin, linezolid, tigecycline and vancomycin while *H. influenzae* was susceptible to most antimicrobial agents in the testing panel (excluding ampicillin).

**Electronic supplementary material:**

The online version of this article (doi:10.1186/s12941-014-0052-2) contains supplementary material, which is available to authorized users.

## Background

*Streptococcus pneumoniae* and *Haemophilus influenzae* are among the most common causes of pneumonia worldwide [1,2]. According to the most recent guidelines for the treatment of community-acquired pneumonia (CAP) published by the Infectious Diseases Society of America and American Thoracic Society [3], the recommended empiric therapy for inpatient CAP includes a β-lactam (cefotaxime, ceftriaxone or ampicillin-sulbactam) plus either a respiratory fluoroquinolone or azithromycin for intensive care unit (ICU) patients or a respiratory fluoroquinolone or β-lactam plus a macrolide for non-ICU patients. European guidelines are also available [4], while several European countries have established their own policies regarding the treatment of CAP including the UK [5], Sweden [6] and the Netherlands [7]. These guidelines often differ in their treatment recommendations, even when bacteriology is similar between countries [8].

The Tigecycline Evaluation and Surveillance Trial (T.E.S.T.) is a global surveillance study which has been ongoing since 2004. This study was designed to monitor the longitudinal activity of a broad panel of comparator agents, including the glycylcycline antimicrobial tigecycline, against a collection of clinically important pathogens. Tigecycline possesses potent in vitro activity against those pathogens most often responsible for community-acquired pneumonia [9]. In 2009, the US Food and Drug Administration approved tigecycline for the treatment of community-acquired bacterial pneumonia (CABP) caused by *Streptococcus pneumoniae* (penicillin-susceptible isolates), including cases with concurrent bacteremia, *Haemophilus influenzae* (β-lactamase negative isolates), and *Legionella pneumophila* in the USA [10], based in part on results from a pair of Phase III clinical trials comparing the efficacy of tigecycline and levofloxacin in hospitalized patients with CABP [11]. In the current study, we examine susceptibility trends among isolates of *S. pneumoniae* and *H. influenzae* collected from Africa, the Asia/Pacific Rim, Europe, Latin America, the Middle East and North America between 2004 and 2012.

This report updates *S. pneumoniae* and *H. influenzae* data previously reported from T.E.S.T. between 2004 and 2008 [12]. In that report, a total of 6785 *S. pneumoniae* and 6642 *H. influenzae* were analyzed. Penicillin resistance among *S. pneumoniae* ranged from 9.3% in Europe to 25.1% in the Asia-Pacific Rim and β-lactamase producing *H. influenzae* ranged from 8.7% in South Africa to 26.8% in the Asia-Pacific Rim. The MIC_90_s for tigecycline against *S. pneumoniae* and *H. influenzae* were ≤0.12 mg/L and ≤2 mg/L, respectively. Data from that 2004-2008 report are included in the dataset used in this report. This report aims to provide an update to Darabi et al. [12], comparing 2004-2008 data with data collected between 2009 and 2012.

## Methods

### Isolate collection

Between 2004 and 2012, a total of 560 centres globally contributed isolates to the T.E.S.T. study in at least a single study year (Table 1). All centres participating in T.E.S.T. were required to contribute a minimum of 65 Gram-positive and 135 Gram-negative isolates per study year; these were to include at least 15 isolates of both *S. pneumoniae* and *H. influenzae* annually. North America and Europe were the main contributors, with 221 and 196 centres participating in the two regions, respectively.

**Table 1:** **Number of centres contributing** ***S. pneumoniae*** **or** ***H. influenzae*** **isolates between 2004 and 2012**

**Region** ^**a**^	**2004**	**2005**	**2006**	**2007**	**2008**	**2009**	**2010**	**2011**	**2012**	**Total** ^**b**^
**Africa**	1	4	4	6	2	2	0	1	8	16
**Asia/** **Pacific Rim**	7	6	20	24	17	14	12	1	13	48
**Europe**	31	25	50	70	104	112	95	35	108	196
**Latin America**	4	13	22	24	35	29	15	6	25	59
**Middle East**	1	2	3	5	9	9	9	4	7	20
**North America**	70	110	99	92	45	43	32	23	80	221
**Global**	114	160	198	221	212	209	163	70	241	560

^a^Africa = Mauritius, Morocco, South Africa, Tunisia; Asia/Pacific Rim = Australia, China, Hong Kong, India, Malaysia, Pakistan, Philippines, Singapore, South Korea, Taiwan, Thailand; Europe = Austria, Belgium, Bulgaria, Croatia, Czech Republic, Denmark, Finland, France, Germany, Greece, Hungary, Ireland, Italy, Latvia, Lithuania, Norway, Poland, Portugal, Romania, Slovak Republic, Slovenia, Spain, Sweden, Switzerland, The Netherlands, United Kingdom; Latin America = Argentina, Brazil, Chile, Colombia, Guatemala, Honduras, Jamaica, Mexico, Panama, Venezuela; Middle East = Israel, Jordan, Kuwait, Lebanon, Oman, Saudi Arabia, Turkey; North America = Canada, United States.

^b^Total = the total number of unique centres contributing isolates between 2004 and 2012.

All body sites were considered acceptable sources for isolate collection; a maximum of 25% of isolates in any year could be urinary in origin. Banked or stored isolates were not accepted, nor were duplicate isolates from a single patient. Isolates were collected from 13 different body sites (Table 2); a small number of isolates were collected from medical instruments. Isolates were included into the study if they were considered a clinically relevant causative organism and the probable causative agent of the infection. Patient age, medical history or gender were not considered relevant. Isolate identification was carried out by contributing centres using routine local methodologies.

**Table 2:** **Geographic distribution and culture source** (>**5**% **of total**) **of** ***S. pneumoniae*** **and** ***H. influenzae*** **isolates**

	**2004** **-** **2008**		**2009** **-** **2012**		**2004** **-** **2012**	
	**n**	**%**	**n**	**%**	**n**	**%**
***S. pneumoniae***	n = 8864		n = 5574		n = 14438	
Region						
Africa	156	1.8	65	1.2	221	1.5
Asia/Pacific Rim	689	7.8	296	5.3	985	6.8
Europe	2786	31.4	2983	53.5	5769	40.0
Latin America	771	8.7	397	7.1	1168	8.1
Middle East	182	2.1	278	5.0	460	3.2
North America	4280	48.3	1555	27.9	5835	40.4
Culture source						
Respiratory	4292	48.4	2583	46.3	6875	47.6
Cardiovascular	2614	29.5	1588	28.5	4202	29.1
HEENT	1255	14.2	861	15.4	2116	14.7
Bodily fluids	463	5.2	346	6.2	809	5.6
***H. influenzae***	n = 8732		n = 6038		n = 14770	
Region						
Africa	157	1.8	61	1.0	218	1.5
Asia/Pacific Rim	688	7.9	302	5.0	990	6.7
Europe	3011	34.5	3311	54.8	6322	42.8
Latin America	747	8.6	410	6.8	1157	7.8
Middle East	210	2.4	281	4.7	491	3.3
North America	3919	44.9	1673	27.7	5592	37.9
Culture source						
Respiratory	6340	72.6	4167	69.0	10507	71.1
HEENT	1562	17.9	1163	19.3	2725	18.4
Cardiovascular	439	5.0	363	6.0	802	5.4

Bodily fluids includes abscess/pus, abdominal, bile, cerebrospinal fluid, pericardial, peritoneal, pleural, synovial and tissue; Cardiovascular includes cardiovascular, blood, blood vessels and heart; HEENT includes head, ears, eyes, nose and throat; Respiratory includes respiratory, bronchial brushing, bronchials, bronchoalveolar lavage, endotracheal aspirate, lungs, sinuses, sputum and trachea.

The central repository for all isolates was International Health Management Associates (IHMA, Schaumburg, IL, USA), who were responsible for organism transport and collection, confirmation of isolate identification and the management of a database including all isolate data. Quality control testing was carried out daily using *S. pneumoniae* ATCC 49619, *H. influenzae* ATCC 49247 and *H. influenzae* ATCC 49766. Approximately 10-15% of isolates were randomly tested annually by IHMA to verify isolate identity and MICs; MIC data were used only if daily QC results were within acceptable ranges as published by the Clinical and Laboratory Standards Institute (CLSI) [13].

### Antimicrobial susceptibility testing

All centres were responsible for minimum inhibitory concentration (MIC) testing using broth microdilution methods described by CLSI [14] using either MicroScan® panels (Dade Microscan Inc., West Sacramento, CA, USA) or Sensititre® plates (TREK Diagnostic Systems, East Grinstead, UK). The T.E.S.T. test panel for *S. pneumoniae* included the following antimicrobial agents: amoxicillin-clavulanate, ampicillin, ceftriaxone, imipenem, levofloxacin, linezolid, meropenem, minocycline, penicillin, piperacillin-tazobactam, tigecycline and vancomycin. Azithromycin, clarithromycin, clindamycin and erythromycin were added to the T.E.S.T. panel for *S. pneumoniae* in 2008, with older isolates tested retrospectively. *H. influenzae* MICs were determined using a panel which included amikacin, amoxicillin-clavulanate, ampicillin, cefepime, ceftriaxone, imipenem, levofloxacin, meropenem, minocycline, piperacillin-tazobactam and tigecycline. In 2006, imipenem testing with MicroScan® plates was replaced in the T.E.S.T. panel with meropenem using Sensititre® plates due to imipenem stability problems.

Antimicrobial susceptibility was determined using CLSI-approved breakpoints [13] with the exception of tigecycline, for which US Food and Drug Administration (FDA)-approved breakpoints were used as CLSI breakpoints are not available for tigecycline [15]. Penicillin oral breakpoints (susceptible ≤0.06 mg/L, resistant ≥2 mg/L) were used for *S. pneumoniae* in this study; penicillin-intermediate *S. pneumoniae* (PISP) was defined as a penicillin MIC between 0.12 and 1 mg/L.

The MIC results for 2004-2008 presented here do not exactly match the results presented in Darabi et al. [12] due to retesting of selected isolates from 2004-2008 through the intervening study period or the deletion of untestable isolates from the T.E.S.T. database (most often due to loss of culture viability).

### Antimicrobial resistance determination

β-lactamase production among *H. influenzae* was determined at collecting centres using preferred local methodologies. The central lab, IHMA, retested all *H. influenzae* if the ampicillin results did not agree with the β-lactamase determination. The central laboratory (IHMA) uses the cefinase test [15]. Only 15 β-lactamase-negative, ampicillin-resistant (BLNAR) *H. influenzae* isolates were collected globally during this study; therefore, we have replicated the approach of Darabi et al. [12] here and have included β-lactamase-negative, ampicillin-intermediate (BLNAI) isolates as well. This resulted in a total of 228 BLNAR isolates globally.

### Statistical analysis

A comparison of rates of susceptibility between the two time periods 2004–2008 and 2009–2012 was carried out using the Fisher’s Exact Test. The upper and lower 95% confidence intervals (CI) presented in this manuscript relate to percentage susceptibility. The 95% CI for the percentage susceptible presented in this manuscript were calculated using the SAS exact method for binomial confidence intervals.

## Results

### *S. pneumoniae*

In total, 14438 *S. pneumoniae* isolates were contributed to T.E.S.T. between 2004 and 2012 (Table 2). North America was the largest contributor of *S. pneumoniae* isolates between 2004 and 2008 (48.3%) while Europe contributed the most isolates between 2009 and 2012 (53.5%) (Table 2). The most common isolate sources for *S. pneumoniae* were respiratory (47.6% of all isolates) followed by cardiovascular (29.1%) (Table 2).

Globally, *S. pneumoniae* was highly susceptible to vancomycin (100%), linezolid (>99.9%), tigecycline (99.9%), and levofloxacin (>98.9%) (Additional file 1: Table S1). The global susceptibility of *S. pneumoniae* to most other T.E.S.T. panel agents decreased slightly, often by <1%, between 2004-2008 and 2009-2012. The largest change was observed for minocycline: susceptibility to this agent decreased significantly (p < 0.0001) from 72.1% in 2004-2008 (95% CI 71.2–73.0) to 51.7% in 2009-2012 (95% CI 50.4–53.0). This drop in minocycline susceptibility was observed in all regions, ranging from 15.5% in Europe to 31.7% in Latin America (Additional file 1: Table S1). Conversely, global susceptibility to meropenem and penicillin increased significantly (p < 0.0001) between 2004–2008 and 2009–2012; for meropenem from 79.5% (95% CI 78.4–80.5) to 83.4% (95% CI 82.4–84.3) and for penicillin from 60.0% (95% CI 59.0–61.1) to 64.8% (95% CI 63.5–66.0). Meropenem susceptibility increased in all regions, although the change was only statistically significant in Europe (p < 0.01) where susceptibility increased from 84.9% (95% CI 83.3–86.5) in 2004–2008 to 88.0% (95% CI 86.8–89.2) in 2009–2012. Penicillin susceptibility increased in Africa (11.7%), Asia/Pacific Rim (3.5%), Europe (0.9%) and North America (6.0%) but decreased in Latin America (7.2%) and the Middle East (3.2%). The change was only statistically significant in North America (p < 0.0001) where susceptibility increased from 57.3% (95% CI 55.8–58.8) to 63.3% (95% CI 60.9–65.7).

Some notable regional changes in *S. pneumoniae* susceptibility were reported which did not impact largely on global rates (Additional file 1: Table S1). In Asia/Pacific Rim, decreases in susceptibility of between 8.3% and 16.0% between 2004–2008 and 2009–2012 were seen for amoxicillin-clavulanate (p < 0.001, 95% CI 86.9–91.6; 76.2-85.4), ceftriaxone (p < 0.0001, 95% CI 87.7–92.3; 72.9–82.6), azithromycin (p < 0.01, 95% CI 44.6–53.0; 33.5–45.2), clarithromycin (p < 0.01, 95% CI 44.8–53.1; 33.5–45.2), erythromycin (p < 0.01, 95% CI 44.5–52.8; 33.2–44.8) and clindamycin (p < 0.0001, 95% CI 61.1–69.0; 43.2–55.1). In the Middle East, macrolide susceptibility decreased by approximately 10% although the decrease was not statistically significant.

Macrolide susceptibility was strongly influenced by penicillin non-susceptibility among *S. pneumoniae*. PISP isolates were 17% less susceptible to macrolides than all *S. pneumoniae* globally combined; susceptibility was reduced by approximately 45% among penicillin-resistant *S. pneumoniae* (PRSP) isolates (Additional file 1: Table S1). Macrolide susceptibility among all *S. pneumoniae* varied widely by region in this study, ranging from 45% in Asia/Pacific Rim to 72%–73% in Latin America; macrolide susceptibility among PRSP ranged from 5% in Asia/Pacific Rim to 45% in Latin America.

Ceftriaxone susceptibility among *S. pneumoniae* isolates decreased in all regions between 2004–2008 and 2009–2012, by 3.2% globally (p < 0.0001) from 96.3% (95% CI 95.9–96.7) to 93.1% (95% CI 92.4–93.7) and by as much as 12.1% in the Asia/Pacific Rim [from 90.1% susceptible (95% CI 87.7–92.3) to 78.0% (95% CI 72.9–82.6)]. The decrease in ceftriaxone susceptibility was considerably more pronounced among PRSP isolates: susceptibility dropped by ≥18.8% in 5 of the 6 regions globally and by as much as 35% in Latin America, decreasing from 80.0% (95% CI 71.1–87.2) to 45.0% (95% CI 32.1–58.4) (p < 0.0001), with a global decrease in susceptibility of 21.2% observed between 2004-2008 (78.1% susceptible, 95% CI 75.8–80.3) and 2009-2012 (56.9% susceptible, 95% CI 53.4–60.4) (p < 0.0001) (Additional file 1: Table S1).

Levofloxacin, linezolid, tigecycline and vancomycin retained similar activity against PRSP isolates compared with all *S. pneumoniae* isolates globally. All other antimicrobial agents showed decreases in activity against PRSP, as high as 45% among the macrolides and over 70% among the carbapenems (meropenem and imipenem) (Additional file 1: Table S1). This was most noticeable in Asia/Pacific Rim, where PRSP susceptibility to the macrolides, ceftriaxone, clindamycin and minocycline (5%, 56.4%, 28.4% and 9.5%, respectively) was much lower than global levels (20-22%, 70.1%, 43.4% and 27.6%).

Regarding resistance phenotypes, 3374 (23.4%) were PISP while 2132 (14.8%) were PRSP globally (Additional file 1: Table S1, Table 3). Regionally, PISP levels were highest in Africa (37.6%), Latin America (31.3%) and Middle East (30.7%) during the complete study interval; PISP levels increased by almost 6% in Latin America but decreased by >18% in Africa, although these changes were not statistically significant. The prevalence of PISP decreased globally, from 25.0% (95% CI 24.1-25.9) in 2004-2008 to 20.8% (95% CI 19.7-21.9) in 2009-2012 (p < 0.0001) (Table 3). PRSP isolates were most prevalent in Asia/Pacific Rim (30.1%) and Africa (27.6%) between 2004 and 2012; rates of PRSP increased in both of these regions between 2004-08 and 2009-12 (>4% and >6%, respectively) although the changes were not statistically significant. The prevalence of PRSP was stable globally, decreasing only slightly from 15.0% (95% CI 14.2-15.7) in 2004-2008 to 14.4% (95% CI 13.5-15.4) in 2009-2012 (Table 3).

**Table 3:** **Rates of PISP and PRSP plus β**-**lactamase**-**positive** ***H. influenzae*** **and BLNAR**, **regionally and globally**

	**PISP n (%)**			**PRSP n (%)**			**BL** **-** **Pos HI n (%)**			**BLNAR** ^**a**^ **n (%)**		
	**2004-08**	**2009-12**	**2004-12**	**2004-08**	**2009-12**	**2004-12**	**2004-08**	**2009-12**	**2004-12**	**2004-08**	**2009-12**	**2004-12**
**Africa**	67/156 (42.9)	16/65 (24.6)	83/221 (37.6)	40/156 (25.6)	21/65 (32.3)	61/221 (27.6)	13/157 (8.3)	6/61 (9.8)	19/218 (8.7)	4/157 (2.5)	1/61 (1.6)	5/218 (2.3)
**Asia/** **Pacific Rim**	152/689 (22.1)	42/296 (14.2)	194/985 (19.7)	198/689 (28.7)	98/296 (33.1)	296/985 (30.1)	192/688 (27.9)	83/302 (27.5)	275/990 (27.8)	23/688 (3.3)	12/302 (4.0)	35/990 (3.5)
**Europe**	536/2786 (19.2)	559/2983 (18.7)	1095/5769 (19.0)	295/2786 (10.6)	302/2983 (10.1)	597/5769 (10.3)	436/3011 (14.5)	499/3311 (15.1)	935/6322 (14.8)	56/3011 (1.9)	45/3311 (1.3)	101/6322 (1.6)
**Latin America**	226/771 (29.3)	139/397 (35.0)	365/1168 (31.3)	105/771 (13.6)	60/397 (15.1)	165/1168 (14.1)	154/747 (20.6)	84/410 (20.5)	238/1157 (20.6)	8/747 (1.1)	12/410 (2.9)	20/1157 (1.7)
**Middle East**	54/182 (29.7)	87/278 (31.3)	141/460 (30.7)	43/182 (23.6)	70/278 (25.2)	113/460 (24.6)	42/210 (20.0)	53/281 (18.9)	95/491 (19.3)	1/210 (0.5)	4/281 (1.4)	5/491 (1.0)
**North America**	1180/4280 (27.6)	316/1555 (20.3)	1496/5835 (25.6)	646/4280 (15.1)	254/1555 (16.3)	900/5835 (15.4)	1015/3919 (25.9)	410/1673 (24.5)	1425/5592 (25.5)	40/3919 (1.0)	22/1673 (1.3)	62/5592 (1.1)
**Global**	2215/8864 (25.0)	1159/5574 (20.8)	3374/14438 (23.4)	1327/8864 (15.0)	805/5574 (14.4)	2132/14438 (14.8)	1852/8732 (21.2)	1135/6038 (18.8)	2987/14770 (20.2)	132/8732 (1.5)	96/6038 (1.6)	228/14770 (1.5)

^a^BLNAR includes both β-lactamase negative, ampicillin-resistant and β-lactamase-negative, ampicillin-intermediate isolates.

### *H. influenzae*

A total of 14770 isolates of *H. influenzae* were contributed between 2004 and 2012 as a part of the T.E.S.T. study. North America was the largest contributor of *H. influenzae* isolates over 2004-2008 (44.9%) while Europe contributed the most isolates over 2009-2012 (54.8%) (Table 2). β-lactamase production was reported among 2987 (20.2%) of isolates while 228 (1.5%) isolates were BLNAR (Table 3). *H. influenzae* was highly susceptible (>98%) to most of the agents on the T.E.S.T. panel; the exception to this was ampicillin, for which susceptibility ranged from 68.7% in Asia/Pacific Rim to 89.0% in Africa. Globally, there was very little change in *H. influenzae* antimicrobial susceptibility between 2004-2008 and 2009-2012 (Additional file 1: Table S2). The most common isolate sources were respiratory (71.1%) and head, ears, eyes, nose and throat (18.4%) (Table 2).

The global prevalence of β-lactamase-positive *H. influenzae* decreased significantly (p < 0.001) during this study, from 21.2% (95% CI 20.4-22.1) in 2004-2008 to 18.8% (95% CI 17.8-19.8) in 2009-2012 (Table 3). The prevalence of β-lactamase-positive *H. influenzae* was highest in Asia/Pacific Rim (27.8%) and North America (25.5%) (Table 3). With the exception of ampicillin (to which β-lactamase-positive isolates are resistant), susceptibility among β-lactamase-positive isolates was stable between 2004-2008 and 2009-2012 (Additional file 1: Table S2).

BLNAR isolates were collected from all regions globally and were least common in Middle East (1.0%) and North America (1.1%); prevalence was highest in Asia/Pacific Rim (3.5%). The global incidence of BLNAR remained consistent during this study, at 1.5% in 2004-2008 and 1.6% in 2009-2012 (Table 3). Antimicrobial susceptibility also did not change over the study period (Additional file 1: Table S2).

## Discussion

This report shows that *S. pneumoniae* global susceptibility has remained relatively stable to most antimicrobial agents globally during the period 2004 to 2012. This reflects the findings of the European Antimicrobial Resistance Surveillance Network (EARS-Net), who showed that non-susceptibility among invasive *S. pneumoniae* in Europe has remained stable between 2004 and 2011, although large variations existed between countries [1]. Results from the SENTRY study in the USA between 1998 and 2011 showed increased resistance among *S. pneumoniae* to amoxicillin-clavulanate, penicillin (at MIC ≥4 mg/L) and ceftriaxone (18.9%, 14.8% and 11.7%, respectively), although levofloxacin, linezolid, tigecycline and vancomycin retained 100% activity [16]. Differences between this study and that of Jones et al. [16] may be attributed to the different time period of isolate collection (SENTRY commenced in 1998 compared with the start of TEST in 2004) as well as the source of isolate collection. Also, geography may influence the findings as the current study is a global collection of isolates compared with the USA analysis of Jones et al. [16]. The change in the CLSI breakpoints for *S. pneumoniae* and penicillin in 2008 also mean that caution must be exercised when comparing susceptibility data between studies [17]. The Jones et al. [16] study used an MIC of ≥4 mg/L for penicillin resistance whereas in this T.E.S.T. study we have used the CLSI penicillin oral breakpoints where resistance is defined as an MIC of ≥2 mg/L.

Susceptibility to ceftriaxone and minocycline among *S. pneumoniae* isolates decreased in all the regions; although in the case of ceftriaxone rates of susceptibility remained high. Reasons for these decreases are unknown and warrant further investigation. One factor that may influence susceptibility among *S. pneumoniae* is the use of the seven- and 13-valent pneumococcal conjugate vaccines (PCV7 and PCV13) which have been shown to modify the epidemiology of pneumococcal disease [18]. The *S. pneumoniae* collected as part of T.E.S.T. which have elevated ceftriaxone and minocycline MICs warrant molecular analysis to investigate the influence of these vaccines on the resistance profile of *S. pneumoniae* globally.

The high levels of susceptibility to tigecycline among *S. pneumoniae* and *H. influenzae* reported in the current study mirror recent results presented elsewhere. The Assessing Worldwide Antimicrobial Resistance Evaluation Program (AWARE) reported 100% tigecycline susceptibility among Latin American *S. pneumoniae* isolates from respiratory tract or complicated skin and soft tissue infections [19]; Zhao et al. reported no tigecycline resistance among 5608 Gram-positive isolates collected in China between 2005 and 2010 [20]; Jones et al. observed 100% tigecycline susceptibility among *S pneumoniae* isolates in the USA between 1998 and 2011 [16]; and Nilsson et al. observed good tigecycline activity against *S. pneumoniae* isolates from Northern European countries with a MIC_90_ of 0.125 mg/L [21]. Nilsson et al. also reported a low MIC_90_ (0.25 mg/L) for tigecycline against *H. influenzae*.

The tigecycline susceptibility results presented here for *S. pneumoniae* are different from those presented by Darabi et al. [12] in their discussion of *S. pneumoniae* collected globally between 2004 and 2008 as a part of the T.E.S.T. study. In the earlier report, *S. pneumoniae* susceptibility to tigecycline ranged from 87.5% in Latin America to 97.1% in the Middle East; in the current report, tigecycline susceptibility was ≥99.8% in all global regions. These changes are likely due to the MIC re-testing of several hundred reportedly tigecycline non-susceptible pneumococci since the publication of Darabi et al. [12]. Re-testing occurred because of the determination that lysed sheep blood in the testing media results in artificially high tigecycline MICs. The re-testing program is carried out by the central laboratory, IHMA, who use un-lysed sheep blood to retest all *S. pneumoniae*. This program began with the retesting of the most recent isolates and then testing backwards to the first isolates received and therefore, some early *S. pneumoniae* MIC results may have been published before all retests were completed. To date, all *S. pneumoniae* with a tigecycline MIC above 0.06 mg/L have retested at or below 0.06 mg/L (data not shown). The difference in results may also be due to the deletion of unevaluable isolates from the T.E.S.T. database. During the production of the dataset used in this manuscript, 236 quarantined *S. pneumoniae* isolates could not be re-evaluated for susceptibility (i.e., isolates which died on transport from collecting centre to the central laboratory [IHMA], were never shipped to IHMA, or could not be resuscitated for retesting) and were thus permanently removed from the T.E.S.T. database (S. Bouchillon, pers com).

As discussed above, in the current study, oral breakpoints (susceptible ≤0.06 mg/L, resistant ≥2 mg/L) have been used for penicillin against *S. pneumoniae* and not the parenteral breakpoints (meningitis: susceptible ≤0.06 mg/L, resistant ≥0.12 mg/L; non-meningitis: susceptible ≤2 mg/L, resistant ≥8 mg/L). Use of these oral breakpoints in the current study suggests that penicillin resistance is high globally (PISP, 23.4%; PRSP, 14.8%), thus oral penicillin treatment of pneumonia caused by *S. pneumoniae* would appear to be ill-advised. However, the occurrence of *S. pneumoniae* isolates with MICs ≥4 mg/L is rare worldwide [22], and the use of high-dose parenteral penicillin is generally effective in the treatment of pneumonia caused by drug-resistant *S. pneumoniae* [23].

*H. influenzae* isolates were highly susceptible to most agents on the T.E.S.T. panel, with global susceptibility ranging from 98.8% (tigecycline) to 99.9% (ceftriaxone, levofloxacin, imipenem and meropenem) during the 2004-2012 interval; the sole exception was ampicillin, to which only 78.3% of isolates were susceptible globally. Regionally, susceptibility in Europe (83.6%) was higher than in North America (73.4%). These regional results compare well with Jones et al., who reported 85.2% and 71.7% ampicillin susceptibility among *H. influenzae* isolates from Europe and the USA, respectively [24]. Global BLNAR levels were low (1.5%) in the current study. Japan is known to have a relatively high prevalence of BLNAR *H. influenzae* [25]; however, no Japanese sites contributed isolates to this study. The USA and Europe, where the majority of T.E.S.T. isolates were collected have lower rates but rates have been reported to be increasing in Europe [26,27]. Studies contemporary with this T.E.S.T. study are scarce making direct comparisons of percentages difficult; however, continued monitoring of these isolates is important, particularly given reports of increasing prevalence.

In the first three years of the T.E.S.T. study, contributing centres in North America were two- to three-fold more numerous than centres from Europe. Since 2008, however, European centres have outnumbered those in North America. This is reflected in the numbers of isolates contributed from these two regions: between 2004 and 2008, 48.3% of *S. pneumoniae* and 44.9% of *H. influenzae* isolates were contributed by centres in North America compared to 31.4% and 34.5%, respectively, by centres in Europe. When the total study period (2004-2012) is considered, *S. pneumoniae* is contributed equally from both regions (40.4% from North America compared to 40.0% from Europe); *H. influenzae* isolates from Europe (42.8%), however, outnumbered those from North America (37.9%). This shift in prevalence from North America to Europe will undoubtedly have influenced overall susceptibility rates observed over the course of this study, as susceptibility was lower in North America than in Europe for most antimicrobial agents between 2004 and 2012. The number of isolates submitted from the other four regions was similar between the two time periods, contributing approximately 19% of isolates. The change in the number of North American and European centres over time reinforces the importance of presentation of regional data from global surveillance studies, where possible. This inconsistency in participating centres over time is an inherent weakness of longitudinal surveillance studies, especially international or global surveillance studies. Thus, as is always the case with data derived from multicentre longitudinal surveillance, the trends in antimicrobial susceptibility reported here must be regarded with some caution.

## Conclusions

*S. pneumoniae* remained highly susceptible to levofloxacin, linezolid, tigecycline and vancomycin while *H. influenzae* was susceptible to most antimicrobial agents in the T.E.S.T. panel (excluding ampicillin). Tigecycline was approved for the treatment of community-acquired bacterial pneumonia in the USA by the FDA in 2009. The results presented in this report show that the in vitro susceptibility of *S. pneumoniae* and *H. influenzae*, two important pathogens which are commonly associated with community-associated pneumonia, has remained high globally in the period since this approval was granted. Tigecycline may thus continue to be an important tool in the armamentarium of physicians for the treatment of CABP.

## Additional file

Additional file 1: Table S1. Antimicrobial susceptibility (MIC_90_ [mg/L] and % susceptibility [%S]) among *S. pneumoniae*, PISP and PRSP isolates. **Table S2.** Antimicrobial susceptibility (MIC_90_ [mg/L] and % susceptibility [%S]) among *H. influenzae*, β-lactamase-positive *H. influenzae* and BLNAR *H. influenzae.*
