# Supplementary material for: Regional and global antimicrobial susceptibility among isolates of Streptococcus pneumoniae and Haemophilus influenzae collected as part of the Tigecycline Evaluation and Surveillance Trial (T.E.S.T.) from 2009 to 2012 and comparison with previous years of T.E.S.T. (2004-2008)
Source: Ann Clin Microbiol Antimicrob. 2014 Nov 7;13:52. doi: 10.1186/s12941-014-0052-2 (PMC4239395; doi:10.1186/s12941-014-0052-2)
Supplement: Additional file 1: Table S1. — Antimicrobial susceptibility (MIC90 [mg/L] and % susceptibility [%S]) among S. pneumoniae, PISP and PRSP isolates. Table S2. Antimicrobial susceptibility (MIC90 [mg/L] and % susceptibility [%S]) among H. influenzae, β-lactamase-positive H. influenzae and BLNAR H. influenzae. [file 12941_2014_52_MOESM1_ESM.doc]

**Table S1.** **Antimicrobial susceptibility** (**MIC_90_** [**mg**/**L**] **and** % **susceptibility** [%**S**]) **among *S***. ***pneumoniae***, **PISP and PRSP isolates**

|  | **2004**-**2008** | | **2009**-**2012** | | **2004**-**2012** | |
| --- | --- | --- | --- | --- | --- | --- |
|  | **MIC_90_** | %**S** | **MIC_90_** | %**S** | **MIC_90_** | %**S** |
| ***S***. ***pneumoniae*** |  |  | |  | |  |
| Africa | n = 156 (50/106; 129)^a^ | | n = 65 (2/63; 57)^a^ | | n = 221 (52/169; 186)^a^ | |
| Amo/cla | 4 | 86.5 | 4 | 84.6 | 4 | 86.0 |
| Amp | 4 | - | 8 | - | 4 | - |
| Azi | ≥128 | 65.1 | 64 | 66.7 | 64 | 65.6 |
| Cro | 1 | 97.4 | 1 | 92.3 | 1 | 95.9 |
| Cla | ≥128 | 65.1 | 64 | 66.7 | ≥128 | 65.6 |
| Cli | ≥128 | 72.1 | ≥128 | 68.4 | ≥128 | 71.0 |
| Ery | ≥128 | 65.1 | 64 | 66.7 | 64 | 65.6 |
| Imi | 0.5 | 52.0 | - | - | 0.5 | 50.0 |
| Lev | 1 | 100 | 1 | 98.5 | 1 | 99.5 |
| Lin | 1 | 99.4 | 1 | 100 | 1 | 99.5 |
| Mer | 1 | 62.3 | 1 | 66.7 | 1 | 63.9 |
| Min | 4 | 79.5 | 8 | 60.0 | 8 | 73.8 |
| Pen | 2 | 31.4 | 4 | 43.1 | 2 | 34.8 |
| Pip/taz | 4 | - | 4 | - | 4 | - |
| Tig | 0.03 | 100 | 0.03 | 100 | 0.03 | 100 |
| Van | 0.5 | 100 | 0.5 | 100 | 0.5 | 100 |
| Asia/Pacific Rim | n = 689 (143/546; 576)^a^ | | n = 296 (1/295; 283)^a^ | | n = 985 (144/841; 859)^a^ | |
| Amo/cla | 4 | 89.4 | 8 | 81.1 | 4 | 86.9 |
| Amp | 4 | - | 8 | - | 4 | - |
| Azi | ≥128 | 48.8 | 64 | 39.2 | ≥128 | 45.6 |
| Cro | 1 | 90.1 | 2 | 78.0 | 2 | 86.5 |
| Cla | ≥128 | 49.0 | ≥128 | 39.2 | ≥128 | 45.8 |
| Cli | ≥128 | 65.1 | ≥128 | 49.1 | ≥128 | 59.8 |
| Ery | ≥128 | 48.6 | ≥128 | 38.9 | ≥128 | 45.4 |
| Imi | 0.5 | 70.6 | - | - | 0.5 | 70.1 |
| Lev | 1 | 98.1 | 1 | 98.3 | 1 | 98.2 |
| Lin | 1 | 100 | 2 | 100 | 1 | 100 |
| Mer | 1 | 64.5 | 1 | 66.1 | 1 | 65.0 |
| Min | ≥16 | 42.8 | ≥16 | 20.9 | ≥16 | 36.2 |
| Pen | 4 | 49.2 | 4 | 52.7 | 4 | 50.3 |
| Pip/taz | 4 | - | 8 | - | 4 | - |
| Tig | 0.06 | 100 | 0.03 | 100 | 0.06 | 100 |
| Van | 0.5 | 100 | 0.5 | 100 | 0.5 | 100 |
| Europe | n = 2786 (747/2039; 2453)^a^ | | n = 2983 (0/2983; 2807)^a^ | | n = 5769 (747/5022; 5260)^a^ | |
| Amo/cla | 1 | 97.0 | 1 | 95.6 | 1 | 96.3 |
| Amp | 2 | - | 2 | - | 2 | - |
| Azi | ≥128 | 71.7 | 64 | 71.9 | 64 | 71.8 |
| Cro | 1 | 97.5 | 1 | 95.4 | 1 | 96.4 |
| Cla | ≥128 | 71.8 | 64 | 72.1 | 64 | 72.0 |
| Cli | ≥128 | 79.8 | ≥128 | 80.5 | ≥128 | 80.2 |
| Ery | ≥128 | 71.3 | 64 | 71.5 | 64 | 71.4 |
| Imi | 0.25 | 85.4 | - | - | 0.25 | 85.4 |
| Lev | 1 | 99.1 | 1 | 99.1 | 1 | 99.1 |
| Lin | 1 | 100 | 1 | 100 | 1 | >99.9 |
| Mer | 0.5 | 84.9 | 0.5 | 88.0 | 0.5 | 86.8 |
| Min | 8 | 67.0 | ≥16 | 51.5 | 8 | 59.0 |
| Pen | 2 | 70.2 | 2 | 71.1 | 2 | 70.7 |
| Pip/taz | 2 | - | 2 | - | 2 | - |
| Tig | 0.06 | 99.9 | 0.03 | >99.9 | 0.03 | 99.9 |
| Van | 0.5 | 100 | 0.5 | 100 | 0.5 | 100 |
| Latin America | n = 771 (146/625; 660)^a^ | | n = 397 (0/397; 378)^a^ | | n = 1168 (146/1022; 1038)^a^ | |
| Amo/cla | 1 | 96.2 | 2 | 92.9 | 2 | 95.1 |
| Amp | 2 | - | 4 | - | 2 | - |
| Azi | 64 | 73.8 | 64 | 72.5 | 64 | 73.3 |
| Cro | 1 | 96.8 | 1 | 90.4 | 1 | 94.6 |
| Cla | 64 | 74.1 | 64 | 72.5 | 64 | 73.5 |
| Cli | 64 | 88.0 | 0.25 | 90.7 | 16 | 89.0 |
| Ery | 64 | 73.9 | 64 | 69.6 | 64 | 72.4 |
| Imi | 0.5 | 84.2 | - | - | 0.5 | 84.2 |
| Lev | 1 | 99.4 | 2 | 98.2 | 1 | 99.0 |
| Lin | 1 | 100 | 2 | 100 | 1 | 100 |
| Mer | 0.5 | 78.6 | 0.5 | 81.1 | 0.5 | 79.5 |
| Min | 8 | 68.0 | ≥16 | 36.3 | 8 | 57.2 |
| Pen | 2 | 57.1 | 2 | 49.9 | 2 | 54.6 |
| Pip/taz | 2 | - | 4 | - | 4 | - |
| Tig | 0.06 | 100 | 0.03 | 100 | 0.06 | 100 |
| Van | 0.5 | 100 | 0.5 | 100 | 0.5 | 100 |
| Middle East | n = 182 (12/170; 159)^a^ | | n = 278 (0/278; 270)^a^ | | n = 460 (12/448; 429)^a^ | |
| Amo/cla | 2 | 90.1 | 4 | 89.9 | 2 | 90.0 |
| Amp | 4 | - | 4 | - | 4 | - |
| Azi | 64 | 75.5 | 64 | 66.3 | 64 | 69.7 |
| Cro | 1 | 91.8 | 1 | 90.3 | 1 | 90.9 |
| Cla | ≥128 | 75.5 | 64 | 65.9 | 64 | 69.5 |
| Cli | ≥128 | 85.5 | ≥128 | 85.6 | ≥128 | 85.5 |
| Ery | 64 | 75.5 | 64 | 65.9 | 64 | 69.5 |
| Imi | 1 | 58.3 | - | - | 1 | 58.3 |
| Lev | 1 | 97.3 | 1 | 97.8 | 1 | 97.6 |
| Lin | 1 | 100 | 1 | 100 | 1 | 100 |
| Mer | 1 | 67.1 | 1 | 70.1 | 1 | 69.0 |
| Min | 8 | 62.1 | ≥16 | 33.1 | ≥16 | 44.8 |
| Pen | 2 | 46.7 | 4 | 43.5 | 4 | 44.8 |
| Pip/taz | 4 | - | 4 | - | 4 | - |
| Tig | 0.03 | 100 | 0.03 | 100 | 0.03 | 100 |
| Van | 0.5 | 100 | 0.5 | 100 | 0.5 | 100 |
| North America | n = 4280 (2051/2229; 3735)^a^ | | n = 1555 (2/1553; 1441)^a^ | | n = 5835 (2053/3782; 5176)^a^ | |
| Amo/cla | 2 | 91.4 | 4 | 87.7 | 2 | 90.4 |
| Amp | 4 | - | 4 | - | 4 | - |
| Azi | ≥128 | 66.3 | 64 | 61.2 | 64 | 64.9 |
| Cro | 1 | 96.7 | 1 | 92.5 | 1 | 95.6 |
| Cla | 64 | 66.7 | 64 | 61.6 | 64 | 65.3 |
| Cli | ≥128 | 83.9 | ≥128 | 81.1 | ≥128 | 83.1 |
| Ery | 64 | 65.8 | 64 | 60.7 | 64 | 64.4 |
| Imi | 0.5 | 70.4 | - | - | 0.5 | 70.3 |
| Lev | 1 | 99.1 | 1 | 99.2 | 1 | 99.1 |
| Lin | 1 | 100 | 1 | 100 | 1 | 100 |
| Mer | 1 | 80.2 | 1 | 81.3 | 1 | 80.6 |
| Min | 4 | 81.0 | ≥16 | 64.8 | 8 | 76.7 |
| Pen | 2 | 57.3 | 4 | 63.3 | 2 | 58.9 |
| Pip/taz | 2 | - | 4 | - | 2 | - |
| Tig | 0.06 | 99.8 | 0.03 | 99.9 | 0.06 | 99.8 |
| Van | 0.5 | 100 | 0.5 | 100 | 0.5 | 100 |
| Global | n = 8864 (3149/5715; 7712)^a^ | | n = 5574 (5/5569; 5236)^a^ | | n = 14438 (3154/11284; 12948)^a^ | |
| Amo/cla | 2 | 93.3 | 2 | 92.0 | 2 | 92.8 |
| Amp | 2 | - | 4 | - | 4 | - |
| Azi | ≥128 | 67.5 | 64 | 66.9 | 64 | 67.2 |
| Cro | 1 | 96.3 | 1 | 93.1 | 1 | 95.1 |
| Cla | ≥128 | 67.8 | 64 | 67.1 | 64 | 67.5 |
| Cli | ≥128 | 81.4 | ≥128 | 79.9 | ≥128 | 80.8 |
| Ery | ≥128 | 67.1 | 64 | 66.3 | 64 | 66.8 |
| Imi | 0.5 | 74.3 | - | - | 0.5 | 74.2 |
| Lev | 1 | 99.0 | 1 | 98.9 | 1 | 99.0 |
| Lin | 1 | 99.9 | 1 | 100 | 1 | >99.9 |
| Mer | 1 | 79.5 | 0.5 | 83.4 | 0.5 | 81.4 |
| Min | 8 | 72.1 | ≥16 | 51.7 | 8 | 64.2 |
| Pen | 2 | 60.0 | 2 | 64.8 | 2 | 61.9 |
| Pip/taz | 2 | - | 4 | - | 2 | - |
| Tig | 0.06 | 99.9 | 0.03 | >99.9 | 0.06 | 99.9 |
| Van | 0.5 | 100 | 0.5 | 100 | 0.5 | 100 |
| **PISP** |  |  |  |  |  |  |
| Africa | n = 67 (29/38; 57)^a^ | | n = 16 (2/14; 13)^a^ | | n = 83 (31/52; 70)^a^ | |
| Amo/cla | 1 | 100 | 1 | 100 | 1 | 100 |
| Amp | 1 | - | 1 | - | 1 | - |
| Azi | 64 | 68.4 | 0.12 | 92.3 | 64 | 72.9 |
| Cro | 1 | 97.0 | 0.5 | 100 | 1 | 97.6 |
| Cla | ≥128 | 68.4 | 0.06 | 92.3 | 64 | 72.9 |
| Cli | ≥128 | 78.9 | 0.06 | 100 | ≥128 | 82.9 |
| Ery | ≥128 | 68.4 | 0.06 | 92.3 | 64 | 72.9 |
| Imi | 0.5 | 41.4 | - | - | 0.5 | 38.7 |
| Lev | 1 | 100 | 1 | 93.8 | 1 | 98.8 |
| Lin | 1 | 98.5 | 1 | 100 | 1 | 98.8 |
| Mer | 0.5 | 78.9 | 0.5 | 85.7 | 0.5 | 80.8 |
| Min | 4 | 85.1 | 4 | 81.3 | 4 | 84.3 |
| Pip/taz | 1 | - | 2 | - | 1 | - |
| Tig | 0.06 | 100 | 0.03 | 100 | 0.06 | 100 |
| Van | 0.5 | 100 | 0.5 | 100 | 0.5 | 100 |
| Asia/Pacific Rim | n = 152 (37/115; 118)^a^ | | n = 42 (1/41; 38)^a^ | | n = 194 (38/156; 156)^a^ | |
| Amo/cla | 1 | 99.3 | 4 | 88.1 | 1 | 96.9 |
| Amp | 2 | - | 4 | - | 2 | - |
| Azi | ≥128 | 42.4 | ≥128 | 31.6 | ≥128 | 39.7 |
| Cro | 0.5 | 98.7 | 1 | 95.2 | 1 | 97.9 |
| Cla | ≥128 | 43.2 | ≥128 | 31.6 | ≥128 | 40.4 |
| Cli | ≥128 | 68.6 | ≥128 | 42.1 | ≥128 | 62.2 |
| Ery | ≥128 | 41.5 | ≥128 | 31.6 | ≥128 | 39.1 |
| Imi | 0.5 | 45.9 | - | - | 0.5 | 44.7 |
| Lev | 1 | 100 | 1 | 100 | 1 | 100 |
| Lin | 1 | 100 | 1 | 100 | 1 | 100 |
| Mer | 0.5 | 78.3 | 0.5 | 78.0 | 0.5 | 78.2 |
| Min | ≥16 | 38.8 | ≥16 | 19.0 | ≥16 | 34.5 |
| Pip/taz | 2 | - | 4 | - | 2 | - |
| Tig | 0.06 | 100 | 0.03 | 100 | 0.06 | 100 |
| Van | 0.5 | 100 | 0.5 | 100 | 0.5 | 100 |
| Europe | n = 536 (154/382; 448)^a^ | | n = 559 (0/559; 528)^a^ | | n = 1095 (154/941; 976)^a^ | |
| Amo/cla | 1 | 98.7 | 1 | 98.9 | 1 | 98.8 |
| Amp | 2 | - | 2 | - | 2 | - |
| Azi | ≥128 | 50.0 | 64 | 47.2 | ≥128 | 48.5 |
| Cro | 1 | 97.4 | 1 | 96.1 | 1 | 96.7 |
| Cla | ≥128 | 50.4 | 64 | 47.5 | ≥128 | 48.9 |
| Cli | ≥128 | 58.9 | ≥128 | 62.5 | ≥128 | 60.9 |
| Ery | ≥128 | 49.6 | ≥128 | 47.0 | ≥128 | 48.2 |
| Imi | 0.5 | 52.6 | - | - | 0.5 | 52.6 |
| Lev | 1 | 98.5 | 1 | 98.4 | 1 | 98.4 |
| Lin | 1 | 99.8 | 1 | 99.8 | 1 | 99.8 |
| Mer | 0.5 | 82.2 | 0.5 | 84.6 | 0.5 | 83.6 |
| Min | ≥16 | 50.9 | ≥16 | 34.3 | ≥16 | 42.5 |
| Pip/taz | 2 | - | 2 | - | 2 | - |
| Tig | 0.06 | 100 | 0.03 | 100 | 0.06 | 100 |
| Van | 0.5 | 100 | 0.5 | 100 | 0.5 | 100 |
| Latin America | n = 226 (37/189; 183)^a^ | | n = 139 (0/139; 129)^a^ | | n = 365 (37/328; 312)^a^ | |
| Amo/cla | 1 | 100 | 1 | 98.6 | 1 | 99.5 |
| Amp | 2 | - | 1 | - | 2 | - |
| Azi | 64 | 65.0 | 64 | 62.8 | 64 | 64.1 |
| Cro | 0.5 | 98.2 | 0.5 | 96.4 | 0.5 | 97.5 |
| Cla | 64 | 65.6 | 64 | 62.8 | 64 | 64.4 |
| Cli | ≥128 | 84.2 | 0.25 | 91.5 | 64 | 87.2 |
| Ery | ≥128 | 65.0 | 64 | 61.2 | 64 | 63.5 |
| Imi | 0.5 | 73.0 | - | - | 0.5 | 73.0 |
| Lev | 1 | 99.1 | 2 | 97.8 | 1 | 98.6 |
| Lin | 1 | 100 | 2 | 100 | 1 | 100 |
| Mer | 0.5 | 74.6 | 0.5 | 84.2 | 0.5 | 78.7 |
| Min | 8 | 67.7 | ≥16 | 37.4 | 8 | 56.2 |
| Pip/taz | 2 | - | 4 | - | 2 | - |
| Tig | 0.06 | 100 | 0.03 | 100 | 0.06 | 100 |
| Van | 0.5 | 100 | 1 | 100 | 0.5 | 100 |
| Middle East | n = 54 (3/51; 48)^a^ | | n = 87 (0/87; 84)^a^ | | n = 141 (3/138; 132)^a^ | |
| Amo/cla | 1 | 98.1 | 2 | 100 | 1 | 99.3 |
| Amp | 2 | - | 2 | - | 2 | - |
| Azi | ≥128 | 70.8 | 64 | 61.9 | 64 | 65.2 |
| Cro | 1 | 98.1 | 1 | 95.4 | 1 | 96.5 |
| Cla | ≥128 | 70.8 | 64 | 60.7 | 64 | 64.4 |
| Cli | ≥128 | 81.3 | ≥128 | 86.9 | ≥128 | 84.8 |
| Ery | ≥128 | 70.8 | 64 | 60.7 | 64 | 64.4 |
| Imi | - | - | - | - | - | - |
| Lev | 2 | 92.6 | 1 | 98.9 | 1 | 96.5 |
| Lin | 1 | 100 | 1 | 100 | 1 | 100 |
| Mer | 0.5 | 66.7 | 0.5 | 79.3 | 0.5 | 74.6 |
| Min | 8 | 59.3 | ≥16 | 36.8 | ≥16 | 45.4 |
| Pip/taz | 2 | - | 4 | - | 4 | - |
| Tig | 0.06 | 100 | 0.03 | 100 | 0.03 | 100 |
| Van | 0.5 | 100 | 0.5 | 100 | 0.5 | 100 |
| North America | n = 1180 (606/574; 1039)^a^ | | n = 316 (2/314; 281)^a^ | | n = 1496 (608/888; 1320)^a^ | |
| Amo/cla | 1 | 99.5 | 1 | 97.5 | 1 | 99.1 |
| Amp | 2 | - | 2 | - | 2 | - |
| Azi | ≥128 | 48.2 | 64 | 38.4 | ≥128 | 46.1 |
| Cro | 0.5 | 99.2 | 0.5 | 97.8 | 0.5 | 98.9 |
| Cla | ≥128 | 48.8 | 64 | 38.1 | 64 | 46.5 |
| Cli | ≥128 | 80.7 | ≥128 | 72.2 | ≥128 | 78.9 |
| Ery | ≥128 | 47.7 | 64 | 38.4 | ≥128 | 45.8 |
| Imi | 0.5 | 44.9 | - | - | 0.5 | 44.7 |
| Lev | 1 | 98.6 | 1 | 99.4 | 1 | 98.8 |
| Lin | 1 | 100 | 1 | 100 | 1 | 100 |
| Mer | 0.5 | 85.7 | 0.5 | 85.7 | 0.5 | 85.7 |
| Min | 8 | 75.0 | ≥16 | 59.2 | 8 | 71.7 |
| Pip/taz | 2 | - | 2 | - | 2 | - |
| Tig | 0.06 | 99.9 | 0.03 | 100 | 0.06 | 99.9 |
| Van | 0.5 | 100 | 0.5 | 100 | 0.5 | 100 |
| Global | n = 2215 (866/1349; 1893)^a^ | | n = 1159 (5/1154; 1073)^a^ | | n = 3374 (871/2503; 2966)^a^ | |
| Amo/cla | 1 | 99.3 | 1 | 98.2 | 1 | 98.9 |
| Amp | 2 | - | 2 | - | 2 | - |
| Azi | ≥128 | 51.1 | 64 | 47.9 | ≥128 | 49.9 |
| Cro | 0.5 | 98.5 | 1 | 96.5 | 0.5 | 97.8 |
| Cla | ≥128 | 51.6 | 64 | 47.9 | ≥128 | 50.3 |
| Cli | ≥128 | 75.1 | ≥128 | 70.2 | ≥128 | 73.3 |
| Ery | ≥128 | 50.7 | 64 | 47.5 | ≥128 | 49.5 |
| Imi | 0.5 | 47.2 | - | - | 0.5 | 47.0 |
| Lev | 1 | 98.6 | 1 | 98.6 | 1 | 98.6 |
| Lin | 1 | 99.9 | 1 | 99.9 | 1 | 99.9 |
| Mer | 0.5 | 81.6 | 0.5 | 84.2 | 0.5 | 82.8 |
| Min | 8 | 65.9 | ≥16 | 41.8 | ≥16 | 57.6 |
| Pip/taz | 2 | - | 2 | - | 2 | - |
| Tig | 0.06 | 99.9 | 0.03 | 100 | 0.06 | 99.9 |
| Van | 0.5 | 100 | 0.5 | 100 | 0.5 | 100 |
| **PRSP** |  |  |  |  |  |  |
| Africa | n = 40 (7/33; 36)^a^ | | n = 21 (0/21; 20)^a^ | | n = 61 (7/54; 56)^a^ | |
| Amo/cla | 4 | 47.5 | 4 | 52.4 | 4 | 49.2 |
| Amp | 8 | - | 8 | - | 8 | - |
| Azi | ≥128 | 27.8 | 64 | 20.0 | ≥128 | 25.0 |
| Cro | 1 | 95.0 | 2 | 76.2 | 2 | 88.5 |
| Cla | ≥128 | 27.8 | 64 | 20.0 | ≥128 | 25.0 |
| Cli | ≥128 | 36.1 | ≥128 | 20.0 | ≥128 | 30.4 |
| Ery | ≥128 | 27.8 | 64 | 20.0 | ≥128 | 25.0 |
| Imi | - | - | - | - | - | - |
| Lev | 1 | 100 | 1 | 100 | 1 | 100 |
| Lin | 1 | 100 | 1 | 100 | 1 | 100 |
| Mer | 1 | 3.0 | 1 | 9.5 | 1 | 5.6 |
| Min | 8 | 50.0 | 8 | 14.3 | 8 | 37.7 |
| Pip/taz | 4 | - | 8 | - | 4 | - |
| Tig | 0.03 | 100 | 0.03 | 100 | 0.03 | 100 |
| Van | 0.5 | 100 | 0.5 | 100 | 0.5 | 100 |
| Asia/Pacific Rim | n = 198 (23/175; 172)^a^ | | n = 98 (0/98; 96)^a^ | | n = 296 (23/273; 268)^a^ | |
| Amo/cla | 8 | 63.6 | ≥16 | 48.0 | 8 | 58.4 |
| Amp | 8 | - | 16 | - | 16 | - |
| Azi | ≥128 | 5.2 | ≥128 | 4.2 | ≥128 | 4.9 |
| Cro | 2 | 66.7 | 8 | 35.7 | 4 | 56.4 |
| Cla | ≥128 | 5.2 | ≥128 | 4.2 | ≥128 | 4.9 |
| Cli | ≥128 | 32.6 | ≥128 | 20.8 | ≥128 | 28.4 |
| Ery | ≥128 | 5.2 | ≥128 | 4.2 | ≥128 | 4.9 |
| Imi | 1 | 4.3 | - | - | 1 | 4.3 |
| Lev | 1 | 95.5 | 1 | 99.0 | 1 | 96.6 |
| Lin | 1 | 100 | 1 | 100 | 1 | 100 |
| Mer | 1 | 3.4 | 2 | 7.1 | 1 | 4.8 |
| Min | ≥16 | 12.6 | ≥16 | 3.1 | ≥16 | 9.5 |
| Pip/taz | 8 | - | 8 | - | 8 | - |
| Tig | 0.03 | 100 | 0.03 | 100 | 0.03 | 100 |
| Van | 0.5 | 100 | 0.5 | 100 | 0.5 | 100 |
| Europe | n = 295 (36/259; 267)^a^ | | n = 302 (0/302; 289)^a^ | | n = 597 (36/561; 556)^a^ | |
| Amo/cla | 8 | 73.9 | 8 | 58.9 | 8 | 66.3 |
| Amp | 8 | - | 8 | - | 8 | - |
| Azi | ≥128 | 26.6 | ≥128 | 28.0 | ≥128 | 27.3 |
| Cro | 2 | 81.4 | 2 | 62.3 | 2 | 71.7 |
| Cla | ≥128 | 27.0 | ≥128 | 28.7 | ≥128 | 27.9 |
| Cli | ≥128 | 43.4 | ≥128 | 43.6 | ≥128 | 43.5 |
| Ery | ≥128 | 26.2 | ≥128 | 24.9 | ≥128 | 25.5 |
| Imi | 0.5 | 0.0 | - | - | 0.5 | 0.0 |
| Lev | 2 | 97.3 | 1 | 98.0 | 1 | 97.7 |
| Lin | 1 | 100 | 1 | 100 | 1 | 100 |
| Mer | 1 | 7.7 | 1 | 10.3 | 1 | 9.1 |
| Min | ≥16 | 31.9 | ≥16 | 18.5 | ≥16 | 25.1 |
| Pip/taz | 4 | - | 8 | - | 8 | - |
| Tig | 0.06 | 100 | 0.03 | 100 | 0.03 | 100 |
| Van | 0.5 | 100 | 0.5 | 100 | 0.5 | 100 |
| Latin America | n = 105 (13/92; 93)^a^ | | n = 60 (0/60; 58)^a^ | | n = 165 (13/152; 151)^a^ | |
| Amo/cla | 4 | 72.4 | 8 | 58.3 | 8 | 67.3 |
| Amp | 8 | - | 16 | - | 8 | - |
| Azi | ≥128 | 43.0 | ≥128 | 48.3 | ≥128 | 45.0 |
| Cro | 2 | 80.0 | 2 | 45.0 | 2 | 67.3 |
| Cla | ≥128 | 43.0 | ≥128 | 48.3 | ≥128 | 45.1 |
| Cli | ≥128 | 66.7 | ≥128 | 67.2 | ≥128 | 66.9 |
| Ery | ≥128 | 43.0 | 64 | 48.3 | ≥128 | 45.1 |
| Imi | 8 | 0.0 | - | - | 8 | 0.0 |
| Lev | 1 | 99.0 | 2 | 95.0 | 2 | 97.6 |
| Lin | 1 | 100 | 1 | 100 | 1 | 100 |
| Mer | 1 | 6.5 | 2 | 11.7 | 1 | 8.6 |
| Min | ≥16 | 41.0 | ≥16 | 16.7 | ≥16 | 32.1 |
| Pip/taz | 4 | - | 8 | - | 8 | - |
| Tig | 0.03 | 100 | 0.03 | 100 | 0.03 | 100 |
| Van | 0.5 | 100 | 0.5 | 100 | 0.5 | 100 |
| Middle East | n = 43 (2/41; 38)^a^ | | n = 70 (0/70; 69)^a^ | | n = 113 (2/111; 107)^a^ | |
| Amo/cla | 8 | 60.5 | 8 | 60.0 | 8 | 60.2 |
| Amp | 8 | - | 8 | - | 8 | - |
| Azi | ≥128 | 57.9 | 64 | 27.5 | 64 | 38.3 |
| Cro | 2 | 67.4 | 2 | 67.1 | 2 | 67.3 |
| Cla | ≥128 | 57.9 | 64 | 27.5 | 64 | 38.3 |
| Cli | ≥128 | 76.3 | ≥128 | 66.7 | ≥128 | 70.1 |
| Ery | ≥128 | 57.9 | 64 | 27.5 | 64 | 38.3 |
| Imi | - | - | - | - | - | - |
| Lev | 1 | 97.7 | 2 | 94.3 | 2 | 95.6 |
| Lin | 1 | 100 | 1 | 100 | 1 | 100 |
| Mer | 1 | 4.9 | 2 | 7.1 | 1 | 6.3 |
| Min | ≥16 | 34.9 | ≥16 | 18.6 | ≥16 | 24.8 |
| Pip/taz | 8 | - | 8 | - | 8 | - |
| Tig | 0.03 | 100 | 0.03 | 100 | 0.03 | 100 |
| Van | 0.5 | 100 | 0.5 | 100 | 0.5 | 100 |
| North America | n = 646 (276/370; 581)^a^ | | n = 254 (0/254; 244)^a^ | | n = 900 (276/624; 825)^a^ | |
| Amo/cla | 8 | 44.3 | 8 | 28.0 | 8 | 39.7 |
| Amp | 8 | - | 16 | - | 8 | - |
| Azi | ≥128 | 18.1 | ≥128 | 11.5 | ≥128 | 16.1 |
| Cro | 2 | 79.4 | 2 | 57.1 | 2 | 73.1 |
| Cla | ≥128 | 18.4 | ≥128 | 11.9 | ≥128 | 16.5 |
| Cli | ≥128 | 44.6 | ≥128 | 32.8 | ≥128 | 41.1 |
| Ery | ≥128 | 17.9 | ≥128 | 11.1 | ≥128 | 15.9 |
| Imi | 1 | 1.4 | - | - | 1 | 1.4 |
| Lev | 1 | 97.8 | 1 | 97.2 | 1 | 97.7 |
| Lin | 1 | 100 | 1 | 100 | 1 | 100 |
| Mer | 1 | 3.0 | 1 | 3.1 | 1 | 3.0 |
| Min | 8 | 38.5 | ≥16 | 22.8 | ≥16 | 34.1 |
| Pip/taz | 4 | - | 8 | - | 8 | - |
| Tig | 0.06 | 99.5 | 0.03 | 99.6 | 0.06 | 99.6 |
| Van | 0.5 | 100 | 0.5 | 100 | 0.5 | 100 |
| Global | n = 1327 (357/970; 1187)^a^ | | n = 805 (0/805; 776)^a^ | | n = 2132 (357/1775; 1963)^a^ | |
| Amo/cla | 8 | 56.6 | 8 | 47.7 | 8 | 53.2 |
| Amp | 8 | - | 16 | - | 8 | - |
| Azi | ≥128 | 21.7 | ≥128 | 21.1 | ≥128 | 21.4 |
| Cro | 2 | 78.1 | 2 | 56.9 | 2 | 70.1 |
| Cla | ≥128 | 21.9 | ≥128 | 21.5 | ≥128 | 21.8 |
| Cli | ≥128 | 45.1 | ≥128 | 40.6 | ≥128 | 43.3 |
| Ery | ≥128 | 21.5 | ≥128 | 19.8 | ≥128 | 20.8 |
| Imi | 1 | 1.4 | - | - | 1 | 1.4 |
| Lev | 1 | 97.5 | 1 | 97.4 | 1 | 97.5 |
| Lin | 1 | 100 | 1 | 100 | 1 | 100 |
| Mer | 1 | 4.7 | 1 | 7.4 | 1 | 6.0 |
| Min | ≥16 | 33.6 | ≥16 | 17.8 | ≥16 | 27.6 |
| Pip/taz | 4 | - | 8 | - | 8 | - |
| Tig | 0.06 | 99.8 | 0.03 | 99.9 | 0.03 | 99.8 |
| Van | 0.5 | 100 | 0.5 | 100 | 0.5 | 100 |

Amo/cla = amoxicillin/clavulanate; Amp = ampicillin; Azi = azithromycin; Cro = ceftriaxone; Cla – clarithromycin; Cli = clindamycin; Ery = erythromycin; Imi = imipenem; Lev = levofloxacin; Lin = linezolid; Mer = meropenem; Min = minocycline; Pen = penicillin; Pip/taz = piperacillin/tazobactam; Tig = tigecycline; Van = vancomycin.

^a^The n values in parentheses represent the numbers of isolates tested against imipenem and meropenem, respectively, followed by the number of isolates tested against macrolides/clindamycin.

**Table S2. Antimicrobial susceptibility** (**MIC_90_** [**mg**/**L**] **and** % **susceptibility** [%**S**]) **among *H***. ***influenzae***, **β**-**lactamase**-**positive *H***. ***influenzae* and BLNAR *H***. ***influenzae***

|  | **2004**-**2008** | | **2009**-**2012** | | **2004**-**2012** | |
| --- | --- | --- | --- | --- | --- | --- |
|  | **MIC_90_** | %**S** | **MIC_90_** | %**S** | **MIC_90_** | %**S** |
| ***H***. ***influenzae*** |  | |  |  | |  |
| Africa | n = 157 (46/111)^b^ | | n = 61 (0/61)^b^ | | n = 218 (46/172)^b^ | |
| Ami | 8 | - | 8 | - | 8 | - |
| Amo/cla | 1 | 100 | 1 | 100 | 1 | 100 |
| Amp | 2 | 89.2 | 2 | 88.5 | 2 | 89.0 |
| Cep | ≤0.5 | 100 | ≤0.5 | 100 | ≤0.5 | 100 |
| Cro | ≤0.06 | 100 | ≤0.06 | 100 | ≤0.06 | 100 |
| Imi | 1 | 100 | - | - | 1 | 100 |
| Lev | 0.03 | 100 | 0.03 | 100 | 0.03 | 100 |
| Mer | 0.25 | 100 | 0.25 | 100 | 0.25 | 100 |
| Min | 1 | 98.7 | 1 | 100 | 1 | 99.1 |
| Pip/taz | ≤0.06 | 99.4 | ≤0.06 | 100 | ≤0.06 | 99.5 |
| Tig | 0.25 | 100 | 0.25 | 95.1 | 0.25 | 98.6 |
| Asia/Pacific Rim | n = 688 (209/479)^b^ | | n = 302 (14/288)^b^ | | n = 990 (223/767)^b^ | |
| Ami | 8 | - | 8 | - | 8 | - |
| Amo/cla | 2 | 99.4 | 2 | 98.7 | 2 | 99.2 |
| Amp | ≥64 | 68.8 | ≥64 | 68.5 | ≥64 | 68.7 |
| Cep | ≤0.5 | 99.7 | ≤0.5 | 100 | ≤0.5 | 99.8 |
| Cro | ≤0.06 | 100 | ≤0.06 | 100 | ≤0.06 | 100 |
| Imi | 1 | 100 | 0.5 | 100 | 1 | 100 |
| Lev | 0.03 | 98.8 | 0.06 | 99.3 | 0.06 | 99.0 |
| Mer | 0.25 | 100 | 0.12 | 100 | 0.25 | 100 |
| Min | 1 | 99.0 | 1 | 99.0 | 1 | 99.0 |
| Pip/taz | ≤0.06 | 100 | ≤0.06 | 99.7 | ≤0.06 | 99.9 |
| Tig | 0.25 | 98.4 | 0.25 | 97.4 | 0.25 | 98.1 |
| Europe | n = 3011 (1039/1972)^b^ | | n = 3311 (31/3280)^b^ | | n = 6322 (1070/5252)^ba^ | |
| Ami | 8 | - | 8 | - | 8 | - |
| Amo/cla | 1 | 99.8 | 1 | 99.7 | 1 | 99.7 |
| Amp | 16 | 83.7 | 16 | 83.6 | 16 | 83.6 |
| Cep | ≤0.5 | 99.4 | ≤0.5 | 99.7 | ≤0.5 | 99.5 |
| Cro | ≤0.06 | 100 | ≤0.06 | 99.8 | ≤0.06 | 99.9 |
| Imi | 1 | 100 | 1 | 96.8 | 1 | 99.9 |
| Lev | 0.03 | 99.9 | 0.03 | 100 | 0.03 | 99.9 |
| Mer | 0.12 | 100 | 0.12 | 99.9 | 0.12 | 99.9 |
| Min | 2 | 97.4 | 1 | 98.9 | 1 | 98.2 |
| Pip/taz | ≤0.06 | 99.9 | ≤0.06 | >99.9 | ≤0.06 | 99.9 |
| Tig | 0.25 | 99.0 | 0.25 | 98.6 | 0.25 | 98.8 |
| Latin America | n = 747 (217/530)^b^ | | n = 410 (8/402)^b^ | | n = 1157 (225/932)^b^ | |
| Ami | 8 | - | 8 | - | 8 | - |
| Amo/cla | 1 | 99.2 | 1 | 99.5 | 1 | 99.3 |
| Amp | 32 | 78.4 | 16 | 76.8 | 16 | 77.9 |
| Cep | ≤0.5 | 99.5 | ≤0.5 | 99.3 | ≤0.5 | 99.4 |
| Cro | ≤0.06 | 100 | 0.12 | 99.3 | ≤0.06 | 99.7 |
| Imi | 1 | 100 | - | - | 1 | 100 |
| Lev | 0.03 | 100 | 0.03 | 100 | 0.03 | 100 |
| Mer | 0.12 | 100 | 0.12 | 100 | 0.12 | 100 |
| Min | 1 | 98.8 | 1 | 98.5 | 1 | 98.7 |
| Pip/taz | ≤0.06 | 99.6 | 0.12 | 98.5 | ≤0.06 | 99.2 |
| Tig | 0.25 | 98.5 | 0.25 | 95.9 | 0.25 | 97.6 |
| Middle East | n = 210 (34/176)^b^ | | n = 281 (0/281)^b^ | | n = 491 (34/457)^b^ | |
| Ami | 8 | - | 8 | - | 8 | - |
| Amo/cla | 2 | 100 | 1 | 100 | 1 | 100 |
| Amp | 32 | 79.5 | 32 | 79.7 | 32 | 79.6 |
| Cep | ≤0.5 | 100 | ≤0.5 | 100 | ≤0.5 | 100 |
| Cro | ≤0.06 | 100 | ≤0.06 | 100 | ≤0.06 | 100 |
| Imi | 2 | 100 | - | - | 2 | 100 |
| Lev | 0.03 | 100 | 0.3 | 100 | 0.03 | 100 |
| Mer | 0.25 | 100 | 0.12 | 100 | 0.25 | 100 |
| Min | 2 | 95.2 | 1 | 100 | 1 | 98.0 |
| Pip/taz | ≤0.06 | 100 | ≤0.06 | 99.6 | ≤0.06 | 99.8 |
| Tig | 0.25 | 99.0 | 0.25 | 98.9 | 0.25 | 99.0 |
| North America | n = 3919 (2004/1915)^b^ | | n = 1673 (70/1603)^b^ | | n = 5592 (2074/3518)^b^ | |
| Ami | 8 | - | 8 | - | 8 | - |
| Amo/cla | 1 | 99.8 | 1 | 99.9 | 1 | 99.8 |
| Amp | ≥64 | 73.1 | ≥64 | 74.2 | ≥64 | 73.4 |
| Cep | ≤0.5 | 99.3 | ≤0.5 | 99.4 | ≤0.5 | 99.3 |
| Cro | ≤0.06 | 100 | ≤0.06 | 99.9 | ≤0.06 | 99.9 |
| Imi | 1 | 99.9 | 1 | 100 | 1 | 99.9 |
| Lev | 0.03 | 100 | 0.03 | 100 | 0.03 | 100 |
| Mer | 0.25 | 100 | 0.12 | 99.6 | 0.25 | 99.8 |
| Min | 1 | 99.1 | 1 | 98.4 | 1 | 98.9 |
| Pip/taz | ≤0.06 | 99.8 | ≤0.06 | 99.6 | ≤0.06 | 99.8 |
| Tig | 0.25 | 99.4 | 0.25 | 98.7 | 0.25 | 99.2 |
| Global | n = 8732 (3549/5183)^b^ | | n = 6038 (123/5915)^b^ | | n = 14770 (3672/11098)^b^ | |
| Ami | 8 | - | 8 | - | 8 | - |
| Amo/cla | 1 | 99.7 | 1 | 99.7 | 1 | 99.7 |
| Amp | 32 | 77.3 | 32 | 79.6 | 32 | 78.3 |
| Cep | ≤0.5 | 99.4 | ≤0.5 | 99.6 | ≤0.5 | 99.5 |
| Cro | ≤0.06 | 100 | ≤0.06 | 99.8 | ≤0.06 | 99.9 |
| Imi | 1 | 99.9 | 1 | 99.2 | 1 | 99.9 |
| Lev | 0.03 | 99.9 | 0.03 | 100 | 0.03 | 99.9 |
| Mer | 0.25 | 100 | 0.12 | 99.9 | 0.12 | 99.9 |
| Min | 1 | 98.4 | 1 | 98.8 | 1 | 98.5 |
| Pip/taz | ≤0.06 | 99.8 | ≤0.06 | 99.8 | ≤0.06 | 99.8 |
| Tig | 0.25 | 99.1 | 0.25 | 98.3 | 0.25 | 98.8 |
| **BL**-**Pos *H***. ***influenzae*** |  |  |  |  |  |  |
| Africa | n = 13 (4/9)^b^ | | n = 6 (0/6)^b^ | | n = 19 (4/15)^b^ | |
| Ami | 8 | - | - | - | 8 | - |
| Amo/cla | 4 | 100 | - | - | 4 | 100 |
| Amp | ≥64 | 0.0 | - | - | ≥64 | 0.0 |
| Cep | ≤0.5 | 100 | - | - | ≤0.5 | 100 |
| Cro | ≤0.06 | 100 | - | - | ≤0.06 | 100 |
| Imi | - | - | - | - | - | - |
| Lev | 0.015 | 100 | - | - | 0.03 | 100 |
| Mer | - | - | - | - | 0.25 | 100 |
| Min | 1 | 100 | - | - | 2 | 100 |
| Pip/taz | ≤0.06 | 100 | - | - | ≤0.06 | 100 |
| Tig | 0.25 | 100 | - | - | 0.25 | 100 |
| Asia/Pacific Rim | n = 192 (46/146)^b^ | | n = 83 (6/77)^b^ | | n = 275 (52/223)^b^ | |
| Ami | 8 | - | 8 | - | 8 | - |
| Amo/cla | 4 | 97.9 | 4 | 96.4 | 4 | 97.5 |
| Amp | ≥64 | 0.0 | ≥64 | 0.0 | ≥64 | 0.0 |
| Cep | ≤0.5 | 99.5 | ≤0.5 | 100 | ≤0.5 | 99.6 |
| Cro | ≤0.06 | 100 | ≤0.06 | 100 | ≤0.06 | 100 |
| Imi | 1 | 100 | - | - | 1 | 100 |
| Lev | 0.03 | 96.4 | 0.06 | 100 | 0.06 | 97.5 |
| Mer | 0.25 | 100 | 0.12 | 100 | 0.25 | 100 |
| Min | 2 | 98.4 | 1 | 100 | 1 | 98.9 |
| Pip/taz | ≤0.06 | 100 | ≤0.06 | 100 | ≤0.06 | 100 |
| Tig | 0.25 | 99.5 | 0.25 | 96.4 | 0.25 | 98.5 |
| Europe | n = 436 (149/287)^b^ | | n = 499 (3/496)^b^ | | n = 935 (152/783)^b^ | |
| Ami | 8 | - | 8 | - | 8 | - |
| Amo/cla | 2 | 98.6 | 2 | 98.2 | 2 | 98.4 |
| Amp | ≥64 | 0.5 | ≥64 | 0.0 | ≥64 | 0.2 |
| Cep | ≤0.5 | 99.3 | ≤0.5 | 99.6 | ≤0.5 | 99.5 |
| Cro | ≤0.06 | 100 | ≤0.06 | 99.6 | ≤0.06 | 99.8 |
| Imi | 1 | 100 | - | - | 1 | 100 |
| Lev | 0.03 | 100 | 0.03 | 100 | 0.03 | 100 |
| Mer | 0.12 | 100 | 0.12 | 100 | 0.12 | 100 |
| Min | 2 | 96.3 | 1 | 99.2 | 1 | 97.9 |
| Pip/taz | 0.12 | 99.3 | ≤0.06 | 100 | ≤0.06 | 99.7 |
| Tig | 0.25 | 99.3 | 0.25 | 98.6 | 0.25 | 98.9 |
| Latin America | n = 154 (31/123)^b^ | | n = 84 (3/81)^b^ | | n = 238 (34/204)^b^ | |
| Ami | 8 | - | 8 | - | 8 | - |
| Amo/cla | 2 | 98.7 | 2 | 97.6 | 2 | 98.3 |
| Amp | ≥64 | 0.6 | ≥64 | 1.2 | ≥64 | 0.8 |
| Cep | ≤0.5 | 99.4 | 1 | 97.6 | ≤0.5 | 98.7 |
| Cro | ≤0.06 | 100 | 0.25 | 98.8 | ≤0.06 | 99.6 |
| Imi | 1 | 100 | - | - | 1 | 100 |
| Lev | 0.03 | 100 | 0.03 | 100 | 0.03 | 100 |
| Mer | 0.12 | 100 | 0.12 | 100 | 0.12 | 100 |
| Min | 1 | 98.1 | 1 | 97.6 | 1 | 97.9 |
| Pip/taz | ≤0.06 | 100 | 0.12 | 98.8 | ≤0.06 | 99.6 |
| Tig | 0.25 | 99.4 | 0.25 | 96.4 | 0.25 | 98.3 |
| Middle East | n = 42 (3/39)^b^ | | n = 53 (0/53)^b^ | | n = 95 (3/92)^b^ | |
| Ami | 8 | - | 8 | - | 8 | - |
| Amo/cla | 2 | 100 | 2 | 100 | 2 | 100 |
| Amp | ≥64 | 0.0 | ≥64 | 0.0 | ≥64 | 0.0 |
| Cep | ≤0.5 | 100 | ≤0.5 | 100 | ≤0.5 | 100 |
| Cro | ≤0.06 | 100 | ≤0.06 | 100 | ≤0.06 | 100 |
| Imi | - | - | - | - | - | - |
| Lev | 0.5 | 100 | 0.3 | 100 | 0.03 | 100 |
| Mer | 0.12 | 100 | 0.12 | 100 | 0.12 | 100 |
| Min | 2 | 95.2 | 1 | 100 | 2 | 97.9 |
| Pip/taz | ≤0.06 | 100 | ≤0.06 | 100 | ≤0.06 | 100 |
| Tig | 0.25 | 97.6 | 0.25 | 98.1 | 0.25 | 97.9 |
| North America | n = 1015 (545/470)^b^ | | n = 410 (13/397)^b^ | | n = 1425 (558/867)^b^ | |
| Ami | 8 | - | 8 | - | 8 | - |
| Amo/cla | 2 | 99.3 | 2 | 99.8 | 2 | 99.4 |
| Amp | ≥64 | 0.2 | ≥64 | 0.0 | ≥64 | 0.1 |
| Cep | ≤0.5 | 98.8 | ≤0.5 | 99.0 | ≤0.5 | 98.9 |
| Cro | ≤0.06 | 100 | ≤0.06 | 100 | ≤0.06 | 100 |
| Imi | 1 | 100 | 0.25 | 100 | 1 | 100 |
| Lev | 0.03 | 100 | 0.03 | 100 | 0.03 | 100 |
| Mer | 0.12 | 100 | 0.12 | 99.0 | 0.12 | 99.5 |
| Min | 1 | 98.7 | 1 | 98.3 | 1 | 98.6 |
| Pip/taz | ≤0.06 | 99.8 | ≤0.06 | 99.3 | ≤0.06 | 99.6 |
| Tig | 0.25 | 99.4 | 0.25 | 98.5 | 0.25 | 99.2 |
| Global | n = 1852 (778/1074)^b^ | | n = 1135 (25/1110)^b^ | | n = 2987 (803/2184)^b^ | |
| Ami | 8 | - | 8 | - | 8 | - |
| Amo/cla | 2 | 99.0 | 2 | 98.7 | 2 | 98.9 |
| Amp | ≥64 | 0.3 | ≥64 | 0.1 | ≥64 | 0.2 |
| Cep | ≤0.5 | 99.1 | ≤0.5 | 99.3 | ≤0.5 | 99.2 |
| Cro | ≤0.06 | 100 | ≤0.06 | 99.7 | ≤0.06 | 99.9 |
| Imi | 1 | 100 | 1 | 100 | 1 | 100 |
| Lev | 0.03 | 99.6 | 0.03 | 100 | 0.03 | 99.8 |
| Mer | 0.12 | 100 | 0.12 | 99.6 | 0.12 | 99.8 |
| Min | 1 | 98.0 | 1 | 98.9 | 1 | 98.3 |
| Pip/taz | ≤0.06 | 99.7 | ≤0.06 | 99.6 | ≤0.06 | 99.7 |
| Tig | 0.25 | 99.4 | 0.25 | 98.2 | 0.25 | 98.9 |
| **BLNAR^a^ *H***. ***influenzae*** |  |  |  |  |  |  |
| Asia/Pacific Rim | n = 23 (2/21)^b^ | | n = 12 (0/12)^b^ | | n = 35 (2/33)^b^ | |
| Ami | 8 | - | 8 | - | 8 |  |
| Amo/cla | 4 | 100 | 4 | 91.7 | 4 | 97.1 |
| Cep | 1 | 100 | 1 | 100 | 1 | 100 |
| Cro | 0.12 | 100 | 0.25 | 100 | 0.12 | 100 |
| Imi | - | - | - | - | - | - |
| Lev | 0.03 | 100 | 0.015 | 100 | 0.03 | 100 |
| Mer | 0.25 | 100 | 0.25 | 100 | 0.25 | 100 |
| Min | 1 | 100 | 1 | 100 | 1 | 100 |
| Pip/taz | 0.12 | 100 | ≤0.06 | 100 | 0.12 | 100 |
| Tig | 0.25 | 95.7 | 0.25 | 100 | 0.25 | 97.1 |
| Europe | n = 56 (10/46)^b^ | | n = 45 (1/44)^b^ | | n = 101 (11/90)^b^ | |
| Ami | 8 | - | 8 | - | 8 | - |
| Amo/cla | 4 | 100 | 4 | 97.8 | 4 | 99.0 |
| Cep | 1 | 100 | 1 | 93.3 | 1 | 97.0 |
| Cro | 0.12 | 100 | 0.12 | 100 | 0.12 | 100 |
| Imi | 0.25 | 100 | - | - | 1 | 90.9 |
| Lev | 0.12 | 100 | 0.12 | 100 | 0.12 | 100 |
| Mer | 0.5 | 100 | 0.5 | 97.7 | 0.5 | 98.9 |
| Min | 2 | 92.9 | 1 | 95.6 | 1 | 94.1 |
| Pip/taz | 0.25 | 100 | 0.25 | 97.8 | 0.25 | 99.0 |
| Tig | 0.25 | 100 | 0.25 | 93.3 | 0.25 | 97.0 |
| Latin America | n = = 8 (3/5)^b^ | | n = 12 (0/12)^b^ | | n = 20 (3/17)^b^ | |
| Ami | - | - | 32 | - | 8 | - |
| Amo/cla | - | - | 2 | 100 | 2 | 90.0 |
| Cep | - | - | 1 | 100 | 1 | 100 |
| Cro | - | - | 1 | 100 | 1 | 100 |
| Imi | - | - | - | - | - | - |
| Lev | - | - | 0.25 | 100 | 0.25 | 100 |
| Mer | - | - | 0.25 | 100 | 0.25 | 100 |
| Min | - | - | 1 | 91.7 | 4 | 85.0 |
| Pip/taz | - | - | 1 | 100 | 1 | 95.0 |
| Tig | - | - | 0.25 | 100 | 0.25 | 100 |
| North America | n = 40 (14/26)^b^ | | n = 22 (3/19)^b^ | | n = 62 (17/45)^b^ | |
| Ami | 8 | - | 8 | - | 8 | - |
| Amo/cla | 4 | 97.5 | 4 | 100 | 4 | 98.4 |
| Cep | 1 | 100 | 1 | 95.5 | 1 | 98.4 |
| Cro | ≤0.06 | 100 | 0.5 | 100 | 0.12 | 100 |
| Imi | 1 | 100 | - | - | 1 | 100 |
| Lev | 0.03 | 100 | 0.03 | 100 | 0.03 | 100 |
| Mer | 0.5 | 100 | 0.5 | 94.7 | 0.5 | 97.8 |
| Min | 2 | 97.5 | 2 | 95.5 | 2 | 96.8 |
| Pip/taz | 0.12 | 97.5 | ≤0.06 | 100 | 0.12 | 98.4 |
| Tig | 0.25 | 100 | 0.25 | 100 | 0.25 | 100 |
| Global | n = 132 (31/101)^b^ | | n = 96 (4/92)^b^ | | n = 228 (35/193)^b^ | |
| Ami | 8 | - | 8 | - | 8 | - |
| Amo/cla | 4 | 97.7 | 4 | 97.9 | 4 | 97.8 |
| Cep | 1 | 100 | 1 | 95.8 | 1 | 98.2 |
| Cro | 0.12 | 100 | 0.5 | 100 | 0.25 | 100 |
| Imi | 1 | 100 | - | - | 1 | 97.1 |
| Lev | 0.06 | 100 | 0.06 | 100 | 0.06 | 100 |
| Mer | 0.5 | 100 | 0.5 | 97.8 | 0.5 | 99.0 |
| Min | 2 | 93.2 | 2 | 95.8 | 2 | 94.3 |
| Pip/taz | 0.25 | 97.7 | 0.25 | 99.0 | 0.25 | 98.2 |
| Tig | 0.25 | 99.2 | 0.25 | 96.9 | 0.25 | 98.2 |

Ami = amikacin; Amo/cla = amoxicillin/clavulanate; Amp = ampicillin; Cep = cefepime; Cro = ceftriaxone; Imi = imipenem; Lev = levofloxacin; Mer = meropenem; Min = minocycline; Pip/taz = piperacillin/tazobactam; Tig = tigecycline.

^a^ BLNAR includes both β-lactamase negative, ampicillin-resistant and β-lactamase-negative, ampicillin-intermediate isolates. BLNAR isolates are not detailed here when n < 10 in a region (2004-2012: Africa, n = 5; Middle East, n = 5).

^b^ The n values in parentheses represent the numbers of isolates tested against imipenem and meropenem, respectively.
